# Effect of Finely Ground Limestone and Dolomite on Compression Strength and Reduction Swelling of Vanadium-Titanium Pellets

**DOI:** 10.3390/ma14164433

**Published:** 2021-08-07

**Authors:** Hao Liu, Shihong Peng, Ke Zhang, Yuelin Qin, Fei Meng, Wenchao He, Weiqiang Liu, Min Chen, Lixiang Yan

**Affiliations:** 1 School of Metallurgy and Materials Engineering, Chongqing University of Science and Technology, Chongqing 401331, China; liuhao@cqust.edu.cn (H.L.); m15902303722@163.com (K.Z.); mengfei@cqust.edu.cn (F.M.); hewenchao1217@163.com (W.H.); 2 Value-Added Process and Clean Extraction of Complex Metal Mineral Resources, Chongqing Municipal Key Laboratory of Institutions of Higher Education, Chongqing 401331, China; 3 Process and Systems Engineering Laboratory, Faculty of Science and Engineering, Åbo Akademi University, FI 20500 Åbo, Finland; weiqiang.liu@abo.fi; 4 Department of Chemical and Metallurgical Engineering, School of Chemical Engineering, Aalto University, FI 00076 Aalto, Finland; min.chen@aalto.fi; 5 Chongqing Zhenyan Eco-Friendly Technology, Chongqing 401331, China; yanlixiang0102@163.com

**Keywords:** vanadium titanium pellets, limestone, dolomite, microstructure

## Abstract

Vanadium−titanium magnetite (VTM) is an important raw material for ironmaking under the situation of increasingly demanding scarce resources. To further improve the metallurgical properties of pellets, and to satisfy the requirements of blast furnace slag basicity, finely ground dolomite and limestone have been added to the pellet. In this study, the effect of finely ground dolomite and limestone on the metallurgical properties (green pellet drop strength, cold compression strength, reduction swelling index, and microscopic mineral structure) of VTM pellets were investigated. With the addition of finely ground dolomite and limestone, the drop strength of the green pellet was improved. The effect of adding finely ground limestone was greater than adding finely ground dolomite. Adding more finely ground dolomite and limestone compared to pellets without limestone and dolomite, the cold compression strength was decreased, which was attributed to the decomposition of limestone and dolomite during the induration of pellets. With the addition of dolomite, the reduction swelling index (RSI) increased firstly and then decreased. When the basicity of the pellet was 0.54 to 0.94, the slag phase with the lowest melting point was formed, corresponding to the maximum of the reduction swelling index. For the pellets with added limestone, the reduction swelling of the pellets deteriorated. The reduction index of the pellets increased and reached the maximum (26.6%) at a basicity of 1.54, which belongs to abnormal swelling.

## 1. Introduction

With the rapid development of the Chinese ironmaking industry, the demand on imported iron ore has risen to 85% in recent years [1,2,3]. At the same time, China still has a large amount of unmined vanadium−titanium magnetite (VTM). VTM has been found in reserves in the Panxi district of China, exceeding 9.66 billion ton [4,5,6]. VTM is an important raw material as an additive for ironmaking under the situation of increasingly lacking scarce resources [7]. The large-scale application of VTM is mainly based on the blast furnace and basic oxygen furnace processes [8]. In this process, VTM is fed into a blast furnace mainly in the form of pellets and sintering ore [9,10]. Pellets, as one of the iron-bearing burdens of blast furnaces, present many advantages, including excellent metallurgical performance, low energy consumption in the production process, and fewer pollution emissions compared with using sintering ore. According to China’s pig iron production in 2016 (700 Mt), which was used to calculate the pollutant emissions, it can be estimated that the emissions of dust, CO_2_, SO_2_, and NOx could be reduced by 0.04, 500, 0.2, and 0.35 Mt, respectively, if the 75% sinter was replaced by 60% pellets, including flux pellets [11,12,13,14]. To further improve the metallurgical properties of pellets, and to satisfy the requirements of blast furnace slag basicity, some fluxing materials have been added to the pellet. Among them, dolomite (MgCO_3_ and CaCO_3_) and limestone (CaCO_3_) are the most common additives because of their low cost and abundant reserves [15].

Dwarapudi et al. studied the effect of MgO and basicity on the metallurgical properties and microstructure of hematite and magnetite pellets, and found that with the increase of basicity and the addition of MgO, the reduction degradation index decreased from 40 to about 10, the reduction swelling index decreased from 40 to below 10, and the softening-melting temperature increased from 1100 to 1200 [16,17,18]. Zhu et al. revealed that the Brazilian specularite pellets have abnormal swelling when the pellet basicity ranges from 0.2 to 0.8. Beyond this range, the reduction swelling index of pellets is lower than 20% [19]. Gao et al. investigated the influence of MgO on magnetite pellets through experiments and by employing an unreacted core model, which found that the metallurgical performance of pellets with added MgO was better than for the ordinary acid pellets. In addition, it was further observed that the low-temperature reduction degradation resistance and anti-swelling resistance of the pellets were significantly improved with adding the lightly burnt dolomite [20,21].

Umadevi et al. demonstrated that the reduction degradation index (RDI) of the pellets decreased for the basicity ranges from 0.08 to 0.33, then increased at the basicity varied from 0.33 to 1.15 [22]. Iljana et al. investigated the effects of limestone addition on the pellet metallurgical properties (reducibility, swelling, cracking, softening temperature, etc.) on a laboratory scale. The results showed that the pellets with the addition of limestone had a better reduction degree compared with the pellets without limestone. With the addition of limestone, both the reduction swelling and cracking of the pellets slightly increased, but those properties were able to satisfy the requirements of the raw material quality of the blast furnace [23]. Mandal et al. found that the addition of lime in hematite pellets could produce more calcium−alumina−silicate phases with the basicity from 0 to 2, which was confirmed by the SEM-EDAX analysis [24]. The consolidation mechanism of pellets varies with their basicity. The low basicity pellet mainly relies on oxide bonding, but the high basicity pellet mainly depends on slag bonding [24]. The influence of basicity on the microstructure and consolidation method of pellets is significant. Friel et al. also found that oxide-bonding was the dominant consolidation method for pellets when the basicity was less than 0.8. However, pellets were bonded together by slag-bonded and oxide-bonded methods when the basicity was from 0.8~1.3. In addition, the calcium ferrite-bonded method is predominant when the basicity of the pellet is beyond 1.3. So, it is necessary to improve the calcination temperature of the pellets added with MgO [25].

Meraj et al. found that different MgO flux carriers had different effects on the properties of pellets [26]. He found that pellets with a MgO content of 0.9% could reduce the reduction degradation index to 7.5%, but a little deterioration in the cold compression strength property also occurred. Adding olivine and magnesite fluxes helped the pellet maintain good cold compression strength properties at a lower induration temperature, and it did not reduce the degradation index to a sufficiently low level. Compared with adding magnesite, olivine better improved the pellet performance because of the different gangue contents [26]. Fan et al. revealed that lime and lightly burnt dolomite made the drop strength of the green pellet decrease firstly, and then it increased with the addition of further additives. Adding an appropriate amount of calcium and magnesium fluxes to form a proper amount of liquid phases was beneficial to improve the cold compression strength of the flux pellets, but excessive liquid phases destroyed the cold compression strength of the pellets, because the addition of MgO inhibited the reification crystallization behavior of the flux pellets [27].

Li et al. investigated whether adding burnt lime and dolomite to hematite–magnetite pellets could increase the reduction swelling index of the pellet, and the reduction swelling index of the pellets was reduced when using serpentine as an additive. The CaO accelerated the formation and growth of metallic iron whiskers and led to abnormal swelling of magnetite. Because MgO was able to inhibit the migration of Fe^2+^, the abnormal swelling did not appear for the pellets with added MgO [28].

Tang et al. reported that when the content of MgO in the VTM pellet was increased from 1.14 wt% to 2.40 wt%, the reduction swelling index decreased from 15.2% to 8.6%; however, the cold compression strength of the oxidized pellets decreased dramatically. The cold compression strength was 1985 N for the pellets with 1.14 wt% MgO, which did not satisfy the requirements for blast-furnace production [29].

Based on the above literature reviews, most of the published papers focused on the effects of adding fluxes bearing MgO and CaO on the pelletizing process, cold compression strength, low-temperature reduction degradation, reduction swelling, and mineral phase formation of the pellets. Pellets made by vanadium and titanium fine powder were successfully used in the blast furnace in China in 2009 [30]. The vanadium−titanium pellets showed a better medium-temperature anti-swelling performance than the hematite pellets, and the iron-containing phases were hematite and iron bookie, which formed a hard-reduced magnetite solid solution and limonite, and further deteriorated the high-temperature reducibility of vanadium titanium magnetite pellets [31]. To the best of the authors’ knowledge, there is no literature about the effect of different types of flux added with vanadium−titanium magnetite pellets. To improve the metallurgical properties of vanadium−titanium magnetite pellets, in the present research, efforts were made to understand the effects of adding different amounts of finely ground dolomite and limestone on green pellet formation, green pellet induration in the pelletizing process, and the microstructure of vanadium−titanium magnetite pellets.

## 2. Materials and Methods

### 2.1. Raw Materials

In this paper, vanadium−titanium magnetite (technical grade, Desheng, Sichuan, China), dolomite (technical grade, Desheng, Sichuan, China), and limestone (technical grade, Desheng, Sichuan, China) all experienced a finely grinding process. Bentonite was mixed into two groups of pellets with different basicities.

**Figure 1:**
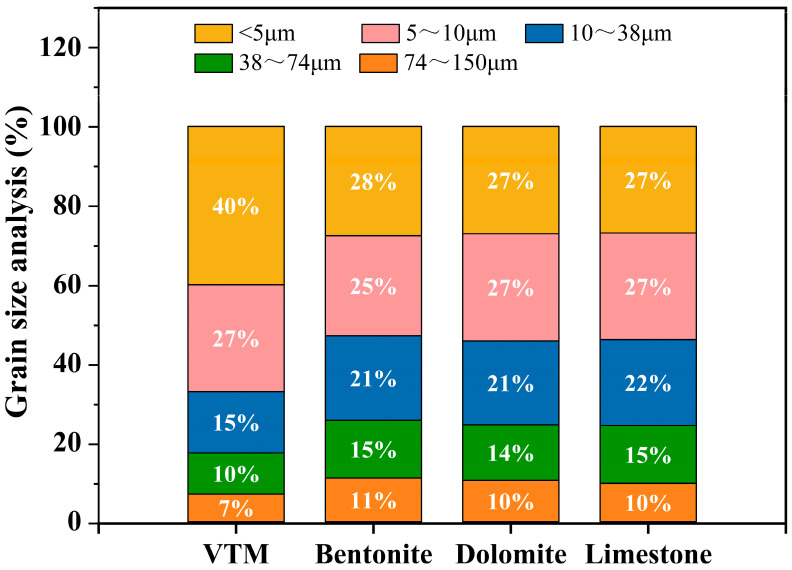
Grain size analysis of the raw materials.

The chemical composition and particle size distribution of the raw materials for making green pellets are shown in Table 1 and Figure 1. The basic physical properties of bentonite are given in Table 2.

### 2.2. Experimental Methods

#### 2.2.1. Preparation of Vanadium−Titanium Magnetite Pellets

**Figure 2:**
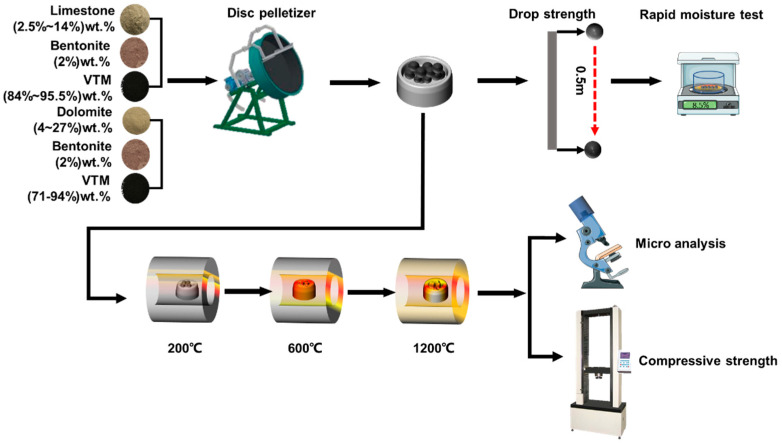
The experimental process of the flux pellets.

The experimental scheme of the pellets is given in Table 3. The experimental process schematic is shown in Figure 2. The main chemical reactions are given in Table 4

Twenty pellets were randomly selected for the drop strength test. The pellets would burst in induration if the green pellet were not dried and preheated. There were three stages in the roasting process. The first stage was to dry the green pellet at 200 °C. The second stage was gradually heated from 200 to 600 °C at a heating rate of 10 °C/min. The preheated green pellets were moved into the muffle furnace, which was heated to 900 °C for the preheating roasting, and then the roasting temperature was increased to 1200 °C at a heating rate of 10 °C/min. Then, the pellets were taken out and cooled in the air to room temperature after roasting for 30 min. The cold compression strength of the roasted pellets was tested by a pellet cold compression strength testing machine WDS-10QT. The air was injected into the muffle furnace during the whole roasting process in order to ensure the oxidation atmosphere in the furnace.

#### 2.2.2. Properties

The green pellet drop strength is an important index to characterize the strength of the green pellet. Ten pellets with a uniform particle size of 10~15 mm were taken and freely dropped from a height of 0.5 m onto a steel plate of 10 mm, one by one. The number of drops when obvious cracks appeared were recorded and the average number of drops was taken as the green pellet drop strength.

Cold compression strength is an important index to characterize whether pellets can be used in production. Ten pellets with a diameter of 10~15 mm were selected. The cold compression strength of the pellets was tested using a pellet cold compression strength testing machine WDS-10QT. The acceleration of the machine should not exceed 10 mm/min. The average cold compression strength of 10 pellets was expressed as the compressive strength.

**Figure 3:**
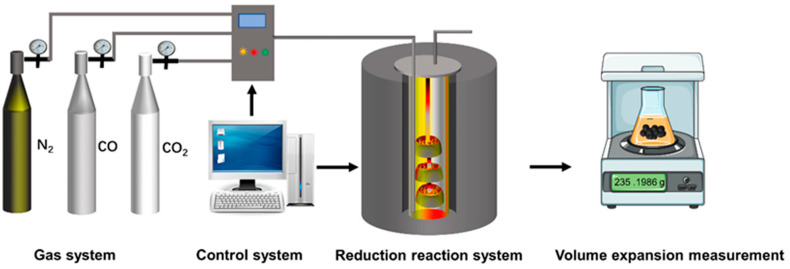
The experimental process of the reduction swelling index test.

The reduction swelling index is an important high-temperature metallurgical indication of the performance of pellets. The experimental process is shown in Figure 3. Eighteen pellets with diameters of 10–12.5 mm were selected and placed in a reaction tube (Ф75 × 800 mm, Chongqing Jinbi Electrical Equipment Co., Ltd., Chongqing, China) and heat at a rate of 10 °C/min. N_2_ with a flow rate of 5 L/min was injected into the reduction tube when the bed temperature reached 200 °C, and the N_2_ flow rate was increased to 15 L/min when the sample temperature reached 900 °C. The heating remained at 900°C for 30 min. The gas in the furnace was changed to the reducing gas, which consisted of 30% CO and 70% N_2_ when the air velocity and temperature of the sample were stable. The volume of pellets before and after reduction was determined by the water immersion method, which was used to calculate the reduction swelling index of the pellets. The calculation formula is below.

λ = (V_2_ − V_1_)/V_1_ (1)

where λ represents the reduction swelling index of the pellets. V_1_ and V_2_ represent the volume of pellets before and after reduction, respectively.

### 2.3. Chemical Analysis and Characterization

The cold compression strength of the samples was tested using a pellet cold compression strength testing machine WDS-10QT. X-ray diffraction (XRD; PANalytical X’Pert Powder XRD (alpha-1), Almelo, The Netherlands) using Cu Ka radiation (40 kV, 40 mA) was used to characterize the mineral phases. The microstructures of the samples were analyzed using a scanning electron microscope (SEM; Tescan MIRA3, Brno, Czech Republic) equipped with an UItraDry Silicon Drift Energy Dispersive X-ray Spectrometer (EDS; Thermo Fisher Scientific, Waltham, MA, USA).

## 3. Results

### 3.1. Moisture and Drop Strength of Green Pellet

**Figure 4:**
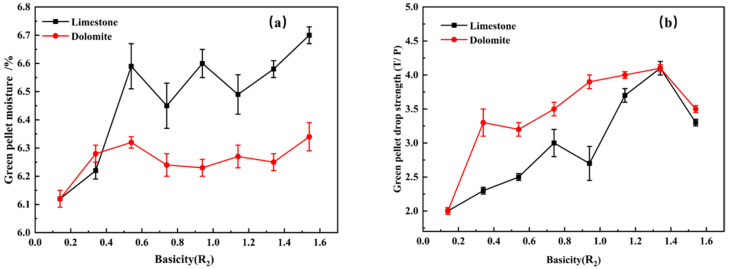
Effect of basicity and flux type on the moisture and drop strength of the green pellets. (**a**) Effect of basicity and flux type on the moisture of the green pellets; (**b**) Effect of basicity and flux type on drop strength of the green pellets.

As shown in Figure 4a, more moisture was needed for the pelletization of finely ground limestone green pellets than for the dolomite green pellets, which indicates that the green pellets with finely ground limestone absorbed more water than the dolomite green pellets during the pelletization. Because the wettability of vanadium−titanium powder was poor, and the proportion of particle size less than 74 μm was 60.993%, and the specific surface area was only 863 cm^2^/g. In addition, the wettability of the finely ground dolomite was weaker than the limestone, so the maximum capillary water content during the pelletization of finely ground dolomite pellets was lower than that of the finely ground limestone pellets, which indicates that dolomite can reduce the strength of the pellets and deteriorate pelletization. As shown in Figure 4b, adding both finely ground limestone and dolomite improved the drop strength of the vanadium−titanium fine powder pellets, and using finely grinding limestone gave a greater effect. In addition, the drop strength of the green pellet decreased when the basicity exceeded 1.34. The addition of finely ground limestone flux significantly improved the water absorption of the raw materials and improved the capillary force of the green pellets.

### 3.2. Cold Compression Strength of Roasted Pellets

**Figure 5:**
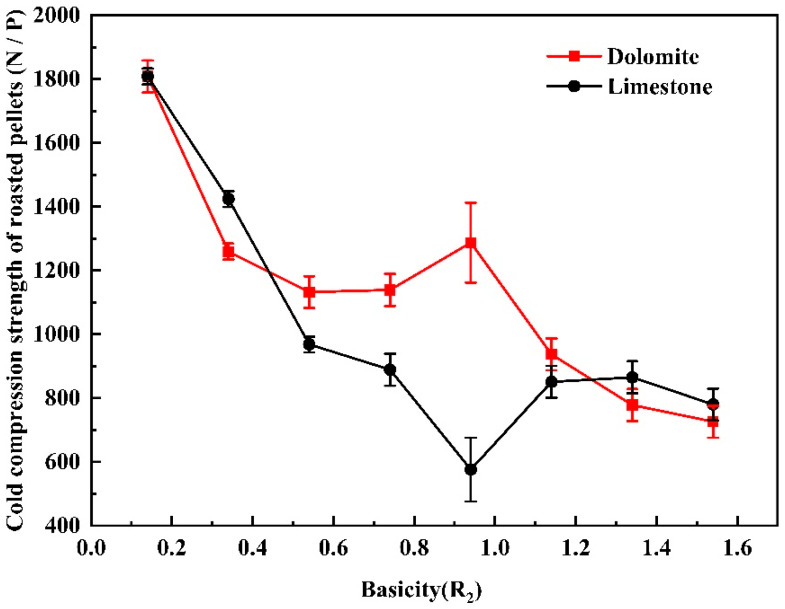
Effect of basicity and flux type on the cold compression strength of the roasted pellets.

**Figure 6:**
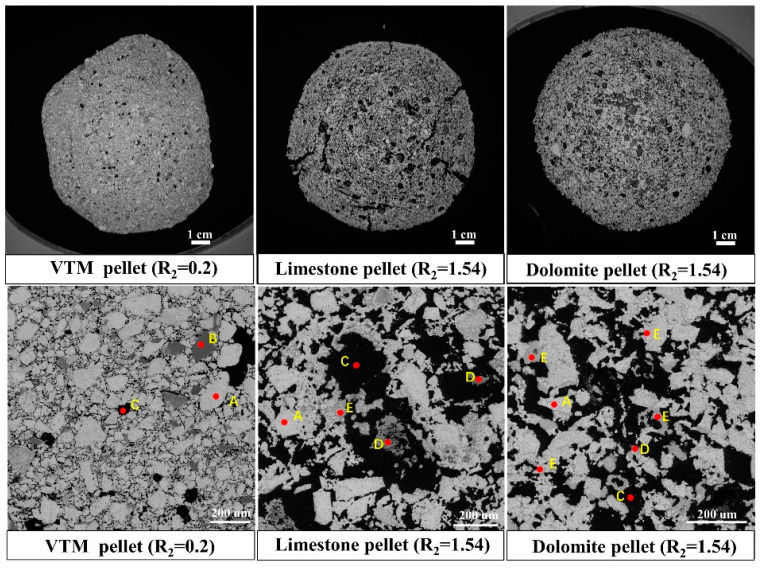
SEM chart of the microstructure of roasted pellets with different types of fluxes. (A) Hematie (Fe_2_O_3_); (B) iron titanium (Fe_0_._975_Ti_0_._025_); (C) pore; (D) andradite titanian (Ca_3_Fe_2_(Si_1_._58_Ti_1_._42_O_12_)); (E) Magnesium Aluminum lron Oxide (MgAl_2_Fe_1.8_O_4_).

Figure 5 shows the effect of the basicity and flux type on the cold compression strength of the roasted pellets. Compared with the pellets without flux, the cold compression strength of the roasted pellets with finely ground limestone and finely ground dolomite decreased significantly with the increase of basicity. At the same basicity, the cold compression strength of the pellets with dolomite flux was greater than that of the pellets with limestone flux when the basicity was between 0.54 to 1.14. The cold compression strength of the finely ground limestone pellets was greater than that of the dolomite pellets when the basicity was beyond 1.34. After adding limestone and dolomite finely ground flux, the cold compression strength of pellets decreased significantly, which failed to satisfy the requirements of blast furnace ironmaking (≥2500 N). Therefore, it was necessary to further adjust the process to improve the cold compression strength of the roasted pellets, for example by increasing the roasting temperature. The consolidation mechanism of the vanadium titanium concentrate pellets was mainly magnetite oxidation and hematite crystallization consolidation. The process of high-temperature roasting of magnetite pellets is an essential oxidation consolidation process. Limestone and dolomite are carbonate minerals. During the roasting process of the pellets, CO_2_ released by carbonate decomposition forms a porous internal structure, which destroys the compactness of pellets and reduces the overall strength [23]. As shown in Figure 6, under the same basicity, the internal structure of the limestone vanadium−titanium pellets was porous and the CO_2_ released by the thermal decomposition of calcium carbonate caused obvious cracks in the pellet sphere, which decreased the strength of the pellets and indicated that adding dolomite was better than limestone.

For the basicity between 0.54 to 1.14, the liquid phase generated by adding calcium-containing materials could improve the spread of Mg^2+^ and Fe^3+^, which was beneficial for magnesium additive mineralization and hematite recrystallization, which then improved the strength of the magnesium pellets [8]. However, the ingredients in this experiment were related to basicity. The contents of MgO and CaO also increased with increasing basicity. The generation of a low melting point liquid phase increased significantly at the same temperature, which weakened the crystallization and consolidation of hematite. The excessive liquid phase penetrated along the grain boundary, destroyed the crystallization of hematite, and reduced the strength of the pellet.

**Figure 7:**
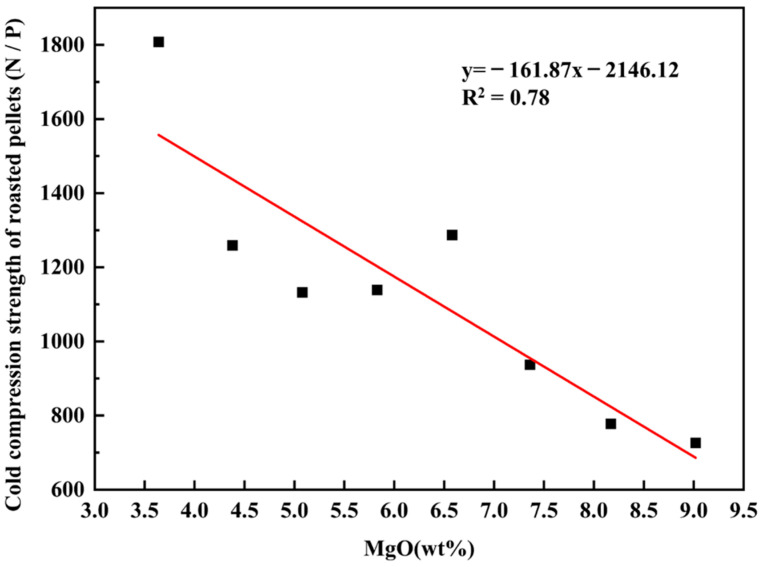
Effect of MgO content on the cold compression strength of roasted pellets.

As shown in Figure 7, the cold compression strength of roasted pellets decreased obviously with the increase of the MgO content. Table 1 illustrates that the vanadium−titanium magnetite is high magnesium ore ((MgO) 3.66 wt.%). With the increase of basicity, the proportion of dolomite continued to increase. In the induration, the two-stage decomposition of dolomite absorbed heat significantly, which inevitably reduced the strength of the preheating pellet [32]. Because of the large amount of Mg^2+^, the structure of magnetite was stabilized and the oxidating magnetite to hematite was hindered, thus weakening the crystallization behavior of hematite, and reducing the strength of the pellets [32]. This is indicated in the XRD phase analysis results. Figure 6 compares the phase compositions of different pellets, i.e., VTM pellet, limestone pellet (at basicity of 1.54), and dolomite pellet (at a basicity of 1.54). Hematite and iron titanium were the main mineral phases of VTM pellets without the addition of flux, and the andradite titanian phase appeared in the main phase with the addition of limestone. The hematite content decreased when adding finely ground limestone. The magnesium−aluminum iron oxide phase was generated in the pellets after reducing the content of finely ground dolomite and the hematite in the pellet. The formation of hematite was significantly reduced with the addition of limestone and dolomite. The fluxes also contributed to the formation of a low melting point mineral phase, namely andradite titanian. Magnesium mainly exists in the form of MgAl_2_Fe_1_._8_O_4_.

### 3.3. Reduction Swelling Index of Pellets

**Figure 8:**
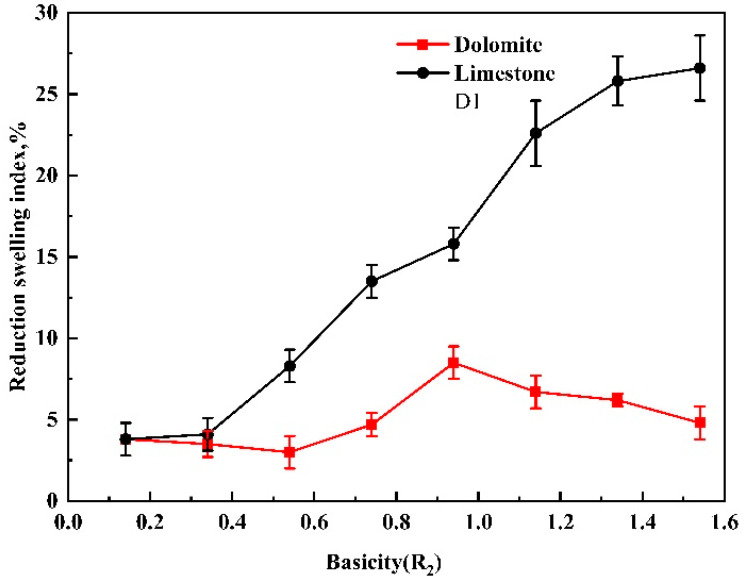
Effect of basicity and flux type on the reduction swelling index of pellets.

As shown in Figure 8, when the basicity increased from 0.14 to 0.54, the reduction swelling index of the dolomite pellets showed a downward trend. When the basicity was 0.54 to 0.94, the reduction swelling index of the pellets increased with the addition of dolomite. However, when the basicity was higher than 0.94, the reduction swelling index of the pellets began to decrease again. With the addition of limestone, the reduction swelling index of the pellets continued to deteriorate, and the highest basicity was 1.54 and the swelling index was 26.6, which belonged to abnormal swelling (RSI > 20%).

**Figure 9:**
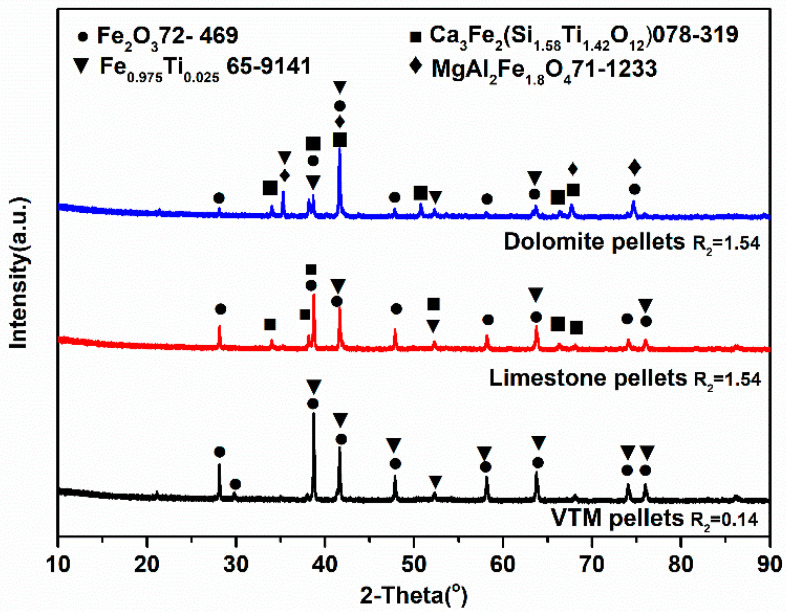
XRD patterns of roasted pellets with different flux types.

As a raw material for blast furnace ironmaking or direct reduction, the pellets entered a reductive atmosphere as they were added into the furnace. The reducing agents (CO, H_2_, and solid carbon) in the furnace reduced the iron oxide by the stepwise reduction reactions, and the ultimate product was metallic iron. In this reduction process, a series of complex physical and chemical changes occurred in the pellets, including the expansion of the pellet volume. Swelling was related to the ability of the gangue or slag phase to withstand the reduction stresses of independent oxide particles. High melting point slag would produce a sufficient bonding strength to limit swelling, but low melting point slag would enhance swelling [18]. When the basicity was between 0.14 to 0.54, the reduction swelling index of the dolomite pellets had a certain decrease because of the high magnesium content in the vanadium−titanium fine powder pellets. With the addition of dolomite, a small amount of slag with a high melting point could be produced to resist the swelling strength. At this time, the low reduction swelling index was mainly attributed to the high porosity of the pellets and a small amount of swelling was occupied by the pore [19]. When the basicity was from 0.54 to 0.94, the swelling was the most serious, because with the increase of the dolomite content in the pellets, in the slag phase, the andradite titanian with the lowest melting point was produced, and the corresponding reduction swelling index (RSI) was the highest, as shown in Figure 9. For this case, the slag phase did not have enough strength to resist the reduction stress. When the basicity was higher than 0.94, a large number of magnesium aluminum iron oxide slag phases with a high melting point were generated in the pellets with the increase in the MgO content. The slag with a high melting point could generate sufficient strength to resist the reduction swelling. For the limestone pellets, the principal effect of adding CaO was to form a low melting point calcium silicate compound, which acted as a bonding agent for the iron oxides during sintering [18]. Low melting calcium silicate and andradite titanian were not enough to produce enough reduction strength to resist the swelling strength generated during reduction. In addition, the addition of limestone increased the liquid content of the pellets, which also deteriorated the reduction swelling. So, the reduction swelling index of limestone pellets continued to increase until reaching the maximum (26.6) at a basicity of 1.54, which belonged to abnormal swelling.

## 4. Conclusions

The effects of adding finely ground dolomite and limestone on the green pellet drop strength, cold compression strength, reduction swelling index, and microscopic mineral structure of vanadium titanium pellets were examined. The following conclusions can be drawn from this work:

- The addition of finely ground dolomite and limestone is conducive to improving the drop strength of the green pellets. The effect of adding finely ground limestone is greater than that of finely ground dolomite.
- With the increase of the content of finely ground dolomite and limestone in the pellet, the cold compression strength of the roasted pellets decreased. When the basicity was between 0.54 to 1.14, under the same basicity, the cold compression strength of the pellets with dolomite flux was greater than that of the pellets with limestone. When the basicity was beyond 1.34, the cold compression strength of finely ground limestone pellets was greater than that of the dolomite pellets.
- With the addition of dolomite, the reduction swelling index of vanadium−titanium pellets increased first and then decreased. When the basicity was from 0.54 to 0.94, the slag with the lowest melting point corresponded to the maximum RSI. For the pellets with added limestone, the reduction swelling of the pellets continued to deteriorate. The reduction index of the pellets increased and reached the maximum (26.6) at a basicity of 1.54, which belonged to abnormal swelling.

## Figures and Tables

**Table 1:** Chemical composition of raw materials/wt %.

	TFe	SiO_2_	CaO	Al_2_O_3_	MgO	TiO_2_	V_2_O_5_	P	S
VTM	54.99	5.53	0.84	4.84	3.66	8.95	0.651	0.007	0.235
Bentonite	1.85	61.68	2.83	13.80	3.02	-	-	0.034	0.036
Dolomite	0.51	4.06	31.05	0.97	18.24	-	-	0.015	0.033
Limestone	0.34	3.72	51.46	1.32	1.44	-	-	0.015	0.102

**Table 2:** Basic physical properties of bentonite.

Moisture Content/%	Colloid Value/[mL·(3 g)^−1^]%	Dilation/(mL·g^−1^)	Liqui Limit/%	Montmorillonite Content/%
10.73	97.5	17.45	303	64.71

**Table 3:** Basic physical properties of bentonite.

	S	Limestone Pellets							Dolomite Pellets						
		L1	L2	L3	L4	L5	L6	L7	D1	D2	D3	D4	D5	D6	D7
Bentonite/%	2.00	2	2	2	2	2	2	2	2	2	2	2	2	2	2
R_2_	0.14	0.34	0.54	0.74	0.94	1.14	1.34	1.54	0.34	0.54	0.74	0.94	1.14	1.34	1.54
MgO/%	3.64	-	-	-	-	-	-	-	4.38	5.08	5.83	6.58	7.36	8.17	9.02

**Table 4:** Main chemical reactions.

Number	Chemical Reaction Equations
(1)	Fe_2_O_3_ + CaO_3_ = CaO·Fe_2_O_3_
(2)	Fe_2_O_3_ + 2CaO = 2CaO·Fe_2_O_3_
(3)	FeO + TiO_2_ = FeO·TiO_2_
(4)	2FeO + TiO_2_ = 2FeO·TiO_2_
(5)	FeO + Al_2_O_3_ = FeO·Al_2_O_3_
(6)	Fe_3_O_4_ + SiO_2_ = 2FeO·SiO_2_
(7)	CaO + TiO_2_ = CaO·TiO_2_
(8)	CaO + SiO_2_ = CaO·SiO_2_
(9)	CaO + SiO_2_ = 3CaO·SiO_2_
(10)	CaO + SiO_2_ = 2CaO·SiO_2_
(11)	CaO + 2SiO_2_ = 3CaO·2SiO_2_
(12)	CaO + 2FeO·TiO2 = CaO·TiO2 +2FeO
(13)	CaO + FeO·TiO_2_ = CaO·TiO_2_ + FeO
(14)	3CaO + 2TiO_2_ = 3CaO·2TiO_2_
(15)	5CaO + 4TiO_2_ = 5CaO·4TiO_2_
(16)	MgO + Al_2_O_3_ = MgO·Al_2_O_3_
(17)	MgO + Fe_2_O_3_ = MgO·Fe_2_O_3_
(18)	CaO·Fe_2_O_3_ +FeO·TiO_2_ = CaO·TiO_2_ + Fe_2_O_3_ + FeO
(19)	CaO·Fe_2_O_3_ + 2FeO·TiO_2_ = CaO·TiO_2_ + Fe_3_O_4_ + FeO
(20)	CaO·Fe_2_O_3_ +TiO_2_ = CaO·TiO_2_ +Fe_2_O_3_
(21)	CaO·Fe_2_O_3_ +SiO_2_ = CaO·SiO_2_ +Fe_2_O_3_

## Data Availability

The study did not report any data.
